# Evaluation of the acute toxicity and 28‐days subacute toxicity of the alcoholic extract from *Ganoderma leucocontextum*

**DOI:** 10.1002/fsn3.3075

**Published:** 2022-09-25

**Authors:** Shizhan Deng, Er‐bu AGA, Hongjun Xie, Hai Xiong, Bengui Ye

**Affiliations:** ^1^ Medical College of Tibet University Lasa China; ^2^ Key Laboratory of Drug‐Targeting and Drug Delivery System of the Education Ministry, Sichuan Engineering Laboratory for Plant‐Sourced Drug and Sichuan Research Center for Drug Precision Industrial Technology West China School of Pharmacy Sichuan University Chengdu China

**Keywords:** acute and subacute toxicities, *Ganoderma leucocontextum*, hematology, histopathology, serum biochemistry

## Abstract

*Ganoderma leucocontextum* is a well‐known traditional medicine in Tibet Autonomous Region, which has benefits, such as anti‐hypoxia, neurotrophic action on nerves, easing coughs and relieving asthma, strengthening the body and prolonging life. However, few research have focused on its negative effects, possibly jeopardizing its safety. The purpose of this study is to evaluate the acute and subacute toxicity of an alcoholic extract from *G. leucocontextum* (GLA) in vivo. The phytochemical characterization analysis showed that alcoholic extract from *G. leucocontextum* were rich in polysaccharides, triterpenoids. Then, in acute oral toxicity, male and female mice from Institute of Cancer Research (ICR) were orally administered with 16 g/kg GLA and were observed for 14 days. In the subacute toxicity, male and female Sprague–Dawley (SD) rats were orally administered with 2, 4, and 8 g/kg doses of GLA for 28 days. There was no death or clinical changes in male and female mice in the acute toxicity test. During the subacute toxicity test, the difference in body weights, food consumption, biochemical and hematological parameters, and organ coefficients between treated and control groups were unrelated to GLA treatment. The obtained data show that the GLA had no significant toxic effects when administered orally to male and female rats in acute and subacute toxicity.

## Graphical abstract

The toxicity of Ganoderma leucocontextum was evaluated for the first time. No toxicological changes were found in acute and subacute toxicity test. Ganoderma leucocontextum showed a good safety profile in animal study.

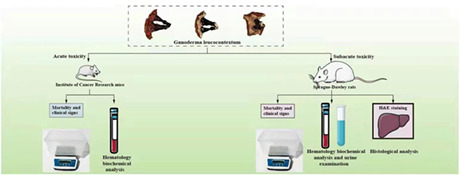

## 1 INTRODUCTION

*Ganoderma leucocontextum* T. H. Li, W. Q. Deng, Sheng H. Wu, Dong M. Wang & H.P.Hu. is a new species of the genus *Ganoderma* found in Tibet in 2014, it is originally from southwestern China and the Tibet region (Li et al., 2015). According to the Tibetan medical classic *Shel Gong Shel Phreng*, *G. leucocontextum* was beneficial for treating hypoxia, trophic action on the nerves, easing cough, relieving asthma, strengthening the body, and prolonging life (Chen et al., 2021).

Because of its ethnopharmacological significance, investigations into the pharmacological potential and phytochemical analyses of this species have been conducted*. G. leucocontextum* included more polysaccharides and triterpenoids than other *Ganoderma* variants, and *G. leucocontextum* contained novel polysaccharides and triterpenoids (Chen et al., 2018; Gao et al., 2020, 2021). Scientific evidence has demonstrated that the triterpenoids from *G. leucocontextum* have anticancer (Li et al., 2019; Liu et al., 2018; Zhao et al., 2016), hypolipidemic effects (Chen et al., 2016; Wang et al., 2015; Zhang et al., 2018), antidiabetics (Wang et al., 2017; Zhang et al., 2017), and neuroprotective effects (Chen et al., 2018). GLP‐1 and GLP‐3, polysaccharides from *G. leucocontextum*, have potent antioxidant and immune boosting effects (Gao et al., 2020, 2021). The potential toxicity of *G. leucocontextum* has been overlooked, and the safe dose has yet to be determined. The toxicity of *G. leucocontextum* aqueous extract was previously assessed (Pan et al., 2021). In the subacute toxicity, no toxicity was shown at the highest dose of 3 g/kg. However, the possible toxicity of other medicinal parts of *G. leucocontextum* was ignored in this previous study, and the design of toxicology studies did not follow the regulatory requirements (China Food and Drug Administration, 2014; OECD, 2002, 2008). The possibility of harm at other concentrations or in alternative types of extracts is not ruled out by these statistics. The recommended daily dosage of *G. leucocontextum* was 6–12 g for adults (60 kg of body weight), but there have not been any essential scientific studies to support it yet (Sichuan Medical Products Administration, 2020). Furthermore, there are no safety studies on the acute or subacute toxicity of *G. leucocontextum* alcoholic extracts, implying that more research is needed to determine the extracts' toxicity. The purpose of this research was to determine the toxicity of *G. leucocontextum* alcoholic extracts in mice and rats using acute and subacute toxicity tests.

## 2 MATERIAL AND METHODS

### 2.1 Plant materials and extraction

The fruiting bodies of *G. leucocontextum* were collected in Linzhi, Tibet, in July 2020, and were identified by associate Prof. Bengui Ye, Department of Pharmacognosy, West China College of Pharmacy, Sichuan University (Chengdu City, Sichuan Province, China). A voucher specimen (V2020031501) has been deposited at the West China College of Pharmacy, Sichuan University.

Searching the literature yielded the extraction protocol for *G. leucocontextum* (Wang et al., 2015). In summary, the dried *G. leucocontextum* powder was soaked in 70% ethanol at a 1:15 (w/v) ratio for 24 h before being refluxed three times at 100°C. The extracted solution was filtered and concentrated under vacuum, and an alcoholic extract of *G. leucocontextum* (GLA, 1466 g) was obtained after freeze‐drying. The w/w extraction rates of GLA were 14.66%, respectively. In other words, 1 g of GLA rough extract equals 6.82 g of raw material.

### 2.2 Phytochemical characterization

Triterpenes and polysaccharides are the major active components of *G. leucocontextum*, hence their quantity in GLA was measured. The content of polysaccharides and total triterpenoids present in GLA was quantified using TU‐1810 UV/Vis (PERSEE, China) and methods specified in the literature (Wu et al., 2020). The anthrone‐sulfuric acid method was used to quantify the content of polysaccharides, with anhydrous glucose as the reference standard (*y* = 4.3719*x* + 0.0049, *R* = .9991). The vanillin‐ice acetic acid method was used to measure total triterpenoids, with oleanolic acid as the reference standard (*y* = 10.605*x* − 0.0045, *R* = .9992).

The phytochemicals contained in the GLA were analyzed using a Dionex Ultimate 3000 UHPLC linked to a Q‐Exactive Plus hybrid quadrupole‐Orbitrap mass spectrometer (Thermo Fisher Scientific, America). The findings were assessed using the Xcalibur data system and Compound Discoverer 3.1 software after samples were dissolved in methanol (1 g/ml) and run entirely at a range of 200–800 m/z (Thermo Fisher Scientific, America). The experiments were conducted in the C‐18 column (Thermo Scientific, America). The first mobile phase (0.1% formic acid) and the second mobile phase (methanol) were on a gradient elution of 0 min: 50% of B, 0–2 min: 50% of B, 3 min: 78% of B, 9 min: and 82% of B, 11 min: 87% of B, 14 min: and 95% of B, 21 min: 100% of B, 23 min: and 50% of B (end of run), at the flow rate of 0.26 ml/min. The identification of peaks was performed by comparing the present results with the retention times (*Rt*) and mass spectrums from the software library.

### 2.3 Test animals

From Chengdu Dossy Experimental Animals CO., LTD. (SCXK2020‐030), we purchased 40 male and female ICR (Institute of Cancer Research) mice weighing 18–22 g, and 80 male and female Sprague–Dawley (SD) rats aged 3 weeks, weighing 50–60 g. All animals were kept in standard laboratory conditions, with 12 h of light/darkness, a relative temperature of 22 ± 2°C, and a humidity of 40–60%. They were provided with food and water at random (Jensen et al., 2013). Five days before the experiment, these animals were acclimatized to the experimental conditions.

### 2.4 Acute toxicity test

Because *G. leucocontextum* has been used in folklore for a long time, it was assumed to be a nontoxic or low‐toxic ethnic medicine, and an acute oral toxicity test was performed using the limit test according to the procedure outlined in National Standards of Food Safety (GB 15193.3‐2014). As a result, the experiments were carried out at the maximum concentration and volume of these extracts.

Twenty male and twenty female ICR mice were randomly divided into two experimental groups: the GLA‐treatment group (16 g/kg GLA) and the negative control group, with 10 male and 10 female mice in each group. Mice in both groups fasted for 12 h before administration to eliminate gastrointestinal feed and drank freely, and then received 16 g/kg GLA via gavage all at once. Three hours after gavage, the diet was resumed, and body weights, general state, development indicators, and toxic reactions were continuously recorded. At the same time, the negative control group was given saline and recorded. After the experiment, all of the animals were fasted for 12 h, anesthesia was administered with 3% sodium pentobarbital, and blood was collected for hematological analysis and serum biochemistry. The CA470 Blood Coagulator Analyzer was used to determine hemoglobin (HGB), hematocrit (HCT), white blood cell count (WBC), red blood cell count (RBC), platelet count (PLT), mean corpuscular volume (MCV), and mean corpuscular hemoglobin (MCH) (MEDONIC, Sweden). A Chemistry Analyzer AU400 was used to measure albumin (ALB), alanine aminotransferase (ALT), aspartate aminotransferase (AST), urea nitrogen (UREA), creatinine (CREA), alkaline phosphatase (ALP), and total cholesterol (CHOL) (Olympus, Tokyo, Japan). Pathological examinations were then performed on the heart, liver, spleen, lung, and kidney. Animals that have been poisoned or euthanized should be dissected as soon as possible to preserve the abnormal organs.

### 2.5 28‐days subacute toxicity test

Because *G. leucocontextum* was nontoxic (LD_50_ > 16 g/kg) in the acute oral toxicity test, the high‐dose group should be increased as much as possible in the subacute toxicity test without affecting the animals' feeding and nutritional balance (Xu et al., 2021). In accordance with the dose design requirements of National Food Safety Standards (GB 15193.22‐2014) and the results of the acute toxicity test, the high‐dose group's dose should be increased as much as possible under the presumption that it will not affect the animals' feeding and nutritional balance, and the interval of the decreasing dose should be 2–4 times. After 5 days of environmental adaptation, 80 SD rats were randomly divided into four groups: high‐dose (8 g/kg), medium‐dose (4 g/kg), low‐dose (2 g/kg), and negative control (0 g/kg). Each group was divided into half males and half females. Saline solutions of GLA with concentrations of 400 mg/ml (maximum solubility), 200, and 100 mg/ml were prepared using saline as the solvent, and the negative control group was given an equal volume of saline with an intragastric volume of 20 ml/kg by weight. Urine samples were collected from each rat at the end of day 28 dosing for analysis. All rats were fasted for 12 h before being anesthetized with 3% sodium pentobarbital and blood was collected in ethylenediaminetetraacetic acid tubes, sodium citrate anticoagulation tubes, and non‐anticoagulated tubes for hematological analysis, and serum biochemistry.

#### 2.5.1 Mortality and clinical signs

During the experiment, the animals were observed twice a day for their coat, skin, eyes, mucous membranes, secretions, excretions, respiratory system, voluntary activities, and behavioral performance. The animals' general clinical manifestations, duration of poisoning, and death were all documented. Weak animals should be isolated, and dead animals should be dissected as soon as possible. Every 3 days, the body weight of each rat was measured and recorded, and the feed consumption of each group was calculated.

#### 2.5.2 Hematological analysis

At the end of the experiment, all rats were fasted for 12 h and blood was obtained from the abdominal aorta under anesthesia for blood index examination. CA470 Blood Coagulator Analyzer (MEDONIC, Sweden) and RAC‐030 Hemagglutination Analyzer (Rayto Life and Analytical Sciences Co., Ltd., Shenzhen, China) were used to determine HGB, HCT, WBC, RBC, PLT, MCV, MCH, lymphocyte ratio (LPR), monocyte ratio (MPR), prothrombin time (PT), activated partial thromboplastin time (APTT), MCHC (mean corpuscular hemoglobin concentration), PCT (Platelet hematocrit), MPV (Mean platelet volume), PDW (Platelet distribution width), and P‐LCR (platelet large cell ratio).

#### 2.5.3 Serum biochemistry

The solidified blood samples were centrifuged at 3500 rpm for 15 min at 4°C in non‐anticoagulated tubes, and the supernatant (serum) was collected. ALT, AST, ALB, UREA, CREA, ALP, total protein (TP), uric acid (UA), glucose (GLU), CHOL, triglycerides (TG), γ‐glutamyl transpeptidase (GGT), creatine kinase (CK), total bilirubin (TBIL), direct bilirubin (DBIL), potassium (K^+^), sodium (Na^+^), and chloride (Cl^−^) were determined using a Chemistry Analyzer AU400 (Olympus, Tokyo, Japan), and the albumin/globulin ratio (A/G) was calculated.

#### 2.5.4 Urine examination

The day before the test, urine was collected for 24 h from each rat using a metabolic cage, and the color of the urine was recorded. A N‐400 Urine analyzer was used to perform qualitative analysis of urine protein, specific gravity, pH, glucose, bilirubin, and occult blood (Dirui Industrial Co., Ltd., Jilin, China).

#### 2.5.5 Macroscopic examination and organ weights

Following blood collection, all rats were subjected to a gross autopsy, which included examination of the body surface, cranial, thoracic, and abdominal cavities, organs, and the location, morphology, color, and size of organs with the naked eye. The organ/body ratio was calculated by weighing the absolute weights of the heart, thymus, adrenal, liver, kidney, spleen, testes, and ovaries. The relative weight of each organ was calculated according to the equation below (Equation [1]).

(1) $$\text{Relative organ weight}=\left[\text{Absolute organ weight}\;\left(g\right)/\text{Body weight}\;\left(g\right)*100\%\right]$$

#### 2.5.6 Histopathological examination

At dissection, the brain, heart, liver, spleen, lung, kidney, pancreas, testis, and ovary were collected from negative control and GLA‐treated animals of each group and fixed in 4% paraformaldehyde fix solution. After fixation, all tissues and organs were dehydrated, embedded, sectioned, stained using hematoxylin and eosin, and observed under a microscope (Xiang et al., 2015).

### 2.6 Statistical analysis

These values are expressed as the means ± standard deviation, and one‐way vascular analysis was performed using SPSS 26.0 statistical software (SPSS Inc., Chicago, IL, USA). A one‐way analysis of variance was performed, followed by a t‐test to assess differences between groups. All treated groups were compared with the control group, * represented *p* < .05, ** represented *p* < .01.

## 3 RESULTS

### 3.1 Phytochemical characterization

**FIGURE 1:**
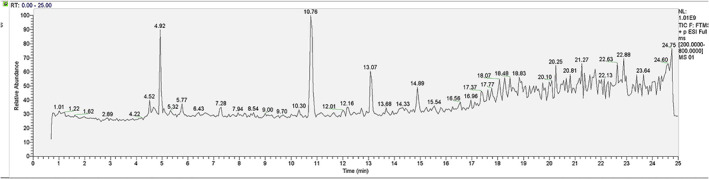
LC/MS total ion chromatogram of the alcoholic extract from *Ganoderma leucocontextum*

The content of total polysaccharides is 15.74 mg of anhydrous glucose equivalent/g of GLA (1.57%), and the content of total triterpenoids is 63.13 mg of oleanolic acid equivalent/g of GLA (6.31%). The retention time (RT) and molecular weight (MW) of the retention peak of the LC–MS were analyzed to identify the phytochemicals. In the present study, the LC/MS chromatogram showed the retention peaks of about 41 compounds. As shown in Figure 1, it was inferred that GLA contains a variety of alkane acids and triterpenes (Table S1).

### 3.2 Acute oral toxicity test

**FIGURE 2:**
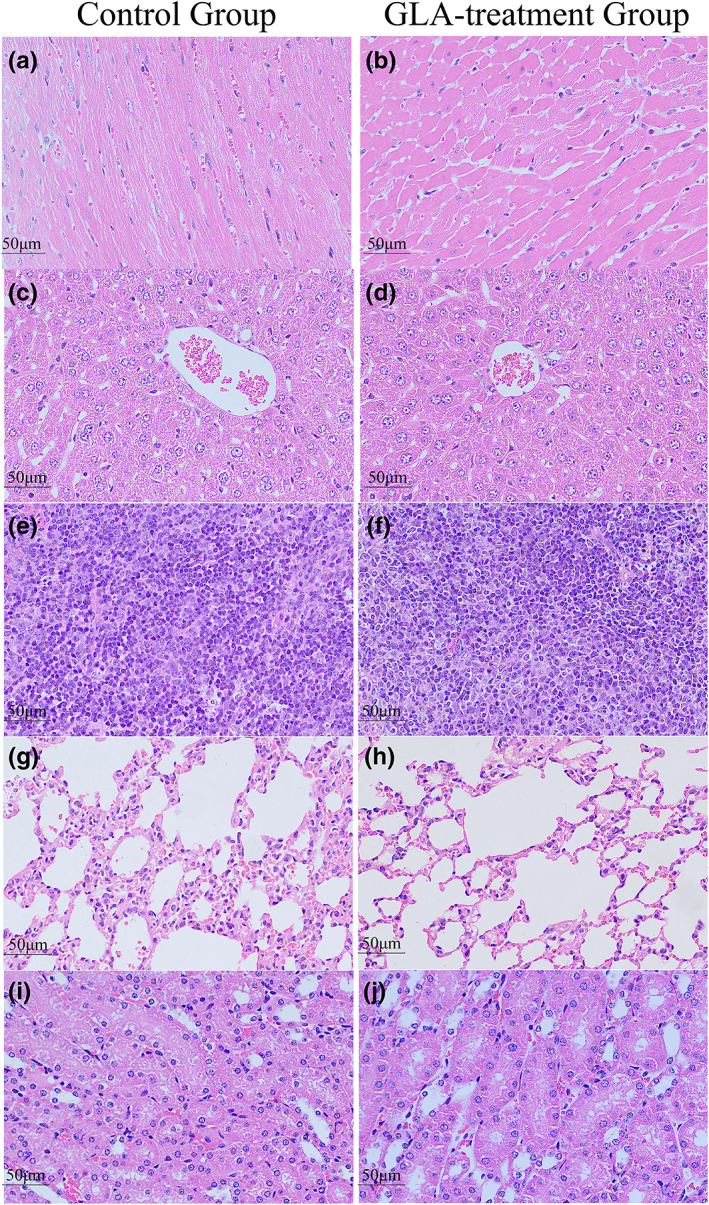
Effect of the alcoholic extract from *Ganoderma leucocontextum* on the microstructures of organs in acute oral toxicity test (H&E: 40×). (a and b) Heart; (c and d) Liver; (e and f) Spleen; (g and h) Lungs; (i and j) Kidney

During the 14‐day observation period, all male and female ICR mice in the negative control and GLA‐treated groups survived. No animals displayed any negative effects or clinical symptoms of poisoning during the study. The body weight change, hematological parameters and serum biochemistry parameters of treated mice were normal (Tables S2–S4). Figure 2 shows that the mice in the GLA‐treated group did not have abnormal pathological examinations of the major organs after autopsies (heart, liver, spleen, lung, and kidney).

### 3.3 28‐days subacute toxicity test

#### 3.3.1 Mortality and clinical signs

**FIGURE 3:**
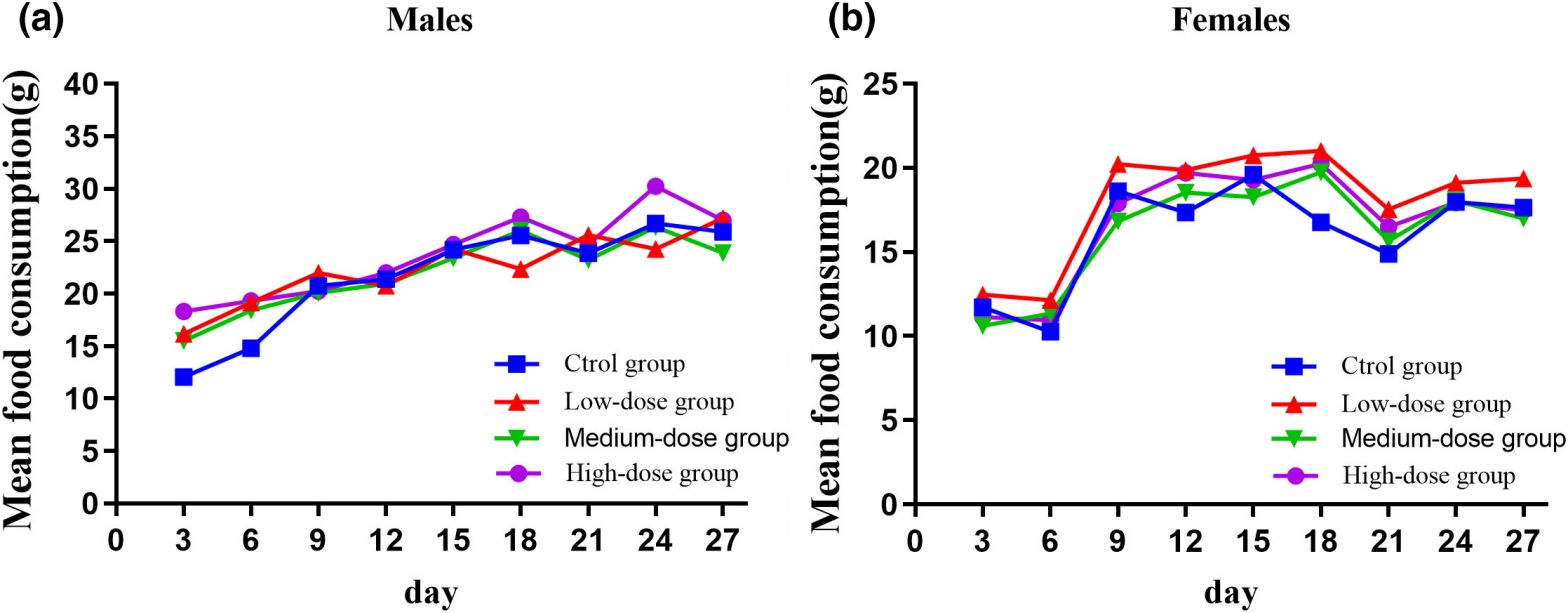
Mean body weight of male and female Sprague–Dawley rats treated with the alcoholic extract from *Ganoderma leucocontextum* during the 28‐days toxicological assessment

**FIGURE 4:**
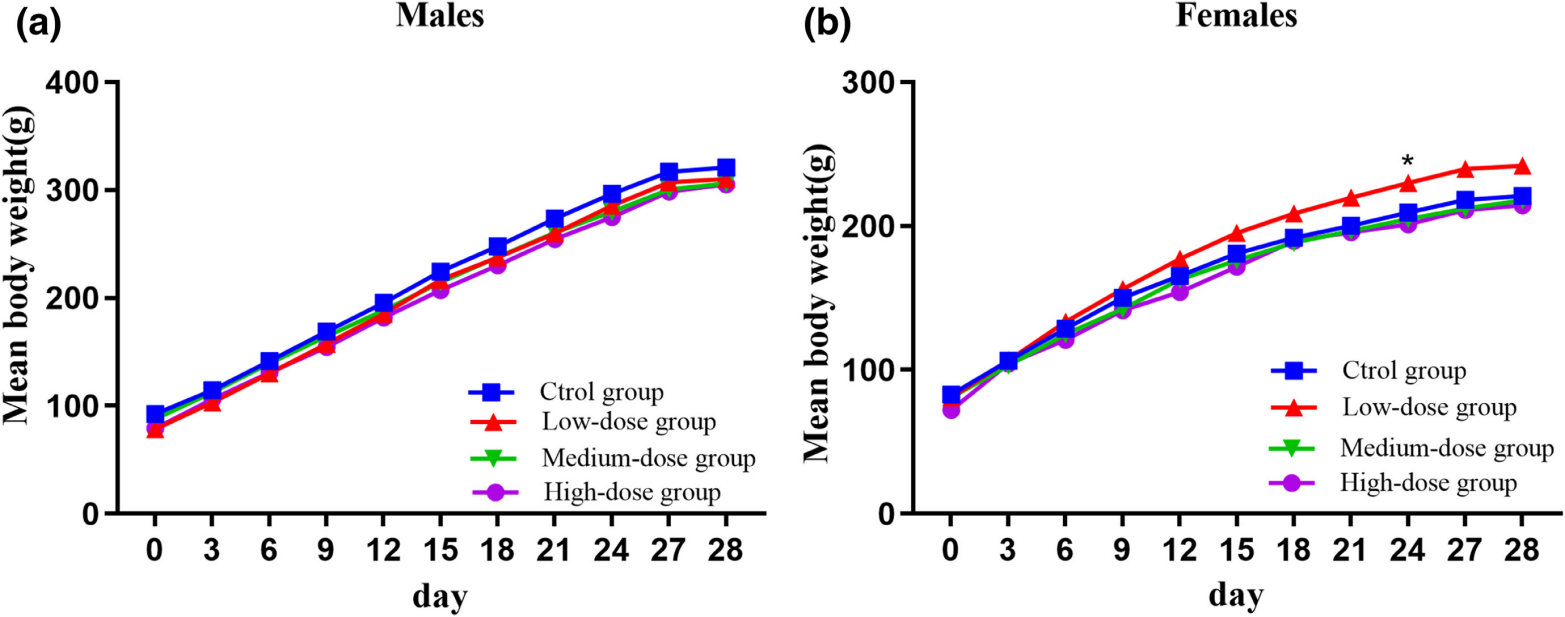
Mean food consumption of male and female Sprague–Dawley rats treated with the alcoholic extract from *Ganoderma leucocontextum* during the 28‐day toxicological assessment

No adverse effects or fatalities were observed during the 28‐day oral GLA treatment (2, 4 and 8 g/kg). Furthermore, when compared to the negative control group, the GLA‐treated animals showed no abnormalities in their behavior or voluntary activity, general behavior, coats, glandular production, breathing, or fecal characteristics. Figure 3 shows the mean body weights of female and male rats. Female rats in the 2 g/kg/d GLA‐treatment group gained significantly more weight from days 21 to 24 when compared with the negative control group (0 g/kg/d) (*p* < .05). Over the period of the experiment, no additional significant differences were found between the treatment groups and the negative control group in both genders. All groups had no effect on the food consumption of male and female rats. In addition, the food consumption of 2 g/kg within male rats decreased in a short time. Nevertheless, the changes were minimal, related to a single time point, and had no toxicological relevance. (Figure 4).

#### 3.3.2 Hematological analysis

In hematological analysis, the results indicated that the WBC in male SD rats was significantly increased at the dose of 4 g/kg/d of GLA (*p* < .05). In females, the LPR of the 8 g/kg/d group decreased significantly when compared with that in the negative control group (*p* < .05), and the PLT was significantly increased in the high‐dose group (*p* < .05). None of the other hematological parameters showed a difference in significance between groups in either gender (Table S5).

#### 3.3.3 Serum biochemistry

Serum biochemistry analysis data are shown in Table S6. In males and females, the level of ALB was significantly increased at the dose of 8 g/kg of GLA groups (*p* < .05). In males, CREA and ALP were significantly increased in mid‐dose (4 g/kg/d) treatment group (*p* < .05) after 28 days of treatment, compared with the negative control group. In females, the level of ALT decreased significantly at the dose of 2 g/kg/d of GLA (*p* < .05). The significant decrease (*p* < .05) of CK and A/G were observed in high‐dose groups after 28 days of treatment, the level of CL^−^ increased significantly at the doses of 8 g/kg of GLA groups (*p* < .05). After 28 days of GLA administration, the other serum biochemistry parameters did not differ from the control group.

#### 3.3.4 Urine examination

In the qualitative urine analysis, there were no obvious differences in urobilinogen, bilirubin, ketone bodies, urine blood, protein, nitrite, leukocytes, glucose, specific gravity, pH, and vitamin C in both female and male rats following a 28‐day oral administration period of GLA (data not shown).

#### 3.3.5 Organ coefficient

After 28 days of treatment with GLA, organ weights were shown in Table S7. In comparison to the control group, the high‐dose GLA‐treated group's kidney relative weight in females was significantly higher (*p* < .05). There were no other significant changes in relative organ weights in any of the treatment groups when compared with the control group. Furthermore, a comprehensive examination of all the major organs of the rats confirmed no abnormalities.

#### 3.3.6 Histopathological analysis

**FIGURE 5:**
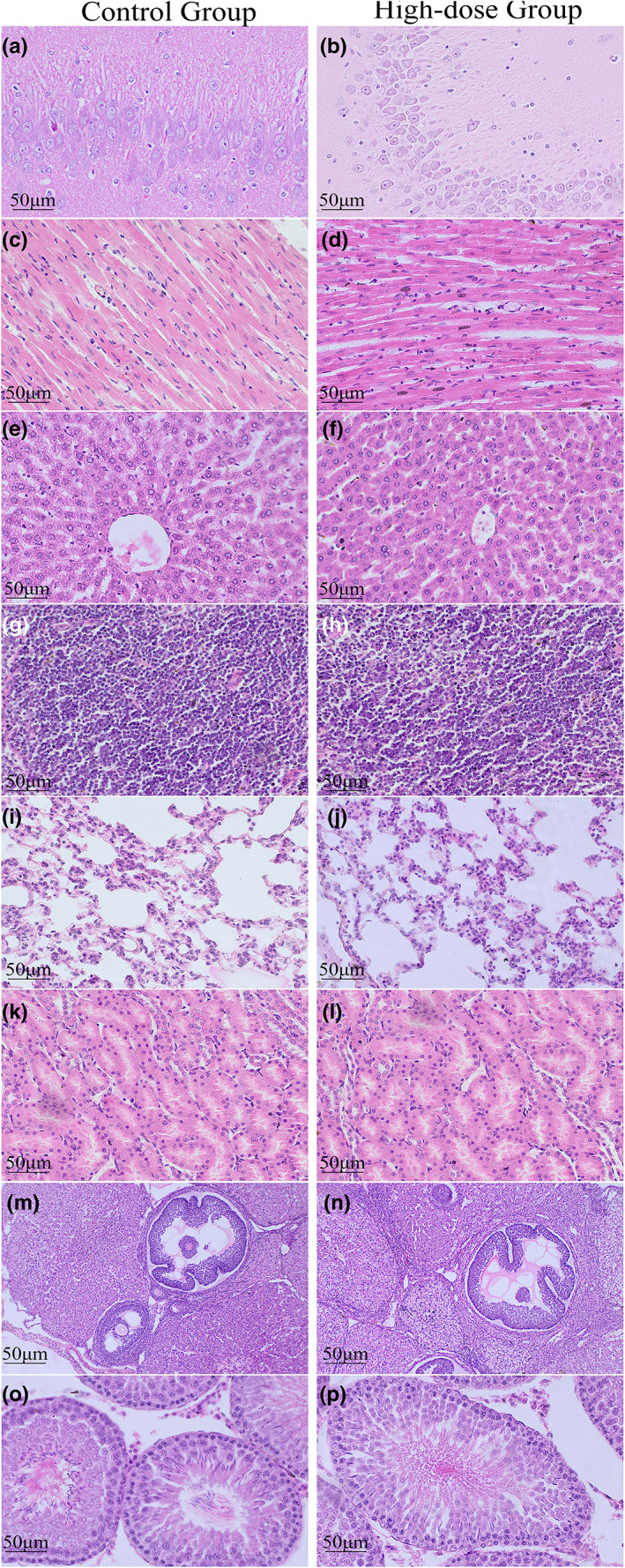
Effect of the alcoholic extract from *Ganoderma leucocontextum* on the microstructures of organs in rats after administration for 28 days (H & E: 40×). (a and b) Brain; (c and d) Heart; (e and f) Liver; (g and h) Spleen; (i and j): Lung; (k and l) Kidney; (m and n): Testis, (o and p) Ovary

Pathological analysis of male and female rats' organs at the end of the 28‐day experiment revealed no detectable abnormalities in the tissues of the brain, heart, liver, spleen, lung, kidney, testis, or ovary. Histopathological examination revealed no abnormalities in microscopic examination in the control or high‐dose groups, and no serious histopathological problems in the high‐dose group, as shown in Figure 5.

## 4 DISCUSSION

*G. leucocontextum* has long been utilized as a medicinal plant in the Tibetan region of China. Although this herb has been used in Tibetan medicine and has a wide range of pharmacological actions, there has been no comprehensive toxicological extensive research on it. The toxicological profile of *G. leucocontextum* needs to be clarified immediately. ICR mice and SD rats were used to test the acute and subacute toxicities of alcoholic extracts of *G. leucocontextum*.

In the acute oral toxicity test, ICR mice were fed 16 g/kg/day of GLA without any side effects or deaths, and at necropsy, no serious organ abnormalities were found. Based on the circumstances of this study, both in male and female rats, the median lethal dose of GLA was predicted to be greater than 16 g/kg body weight. Substances with an oral LD50 greater than 5 g/kg may be considered harmless (Kennedy Jr. et al., 1986). According to the criteria of acute toxic classifications of Ministry of Health, China, 2014, the GLA is actually not toxic (Xu et al., 2021).

In the 28‐day subacute oral toxicity study, GLA caused neither any sign of toxicity nor death in SD rats. Changes in body weight have been used to predict pharmacological and chemical side effects. GLA had the effect of increasing the food consumption of female rats. The body weight of female rats in the low‐dose group was faster than that of the negative control group during the whole experiment period. The body weight and food intake statistical findings showed no specific dosage association or sex responsiveness, indicating that the increases in body weight and food consumption were not thought to have any clinical significance.

The hematological function is amongst the most sensitive indications of the toxicity of drugs, analyzing changes in blood and biochemical parameters are essential (Xiang et al., 2015). Hematological parameters in the treated groups showed alterations, such as a minor increase in WBC, PLT, and a decline in LPR in the 8 g/kg female rats. In histopathological analysis, GLA did not cause any abnormalities in the blood metabolism‐related organs (liver, spleen, or kidney). The alterations were considered to be toxicologically unconnected because they did not depend on dosage or were not reflected by changes in other critical parameters and were within normal physiological limits (Piao et al., 2013; Villas Boas et al., 2018; Zhong‐Ze et al., 2010).

The liver is the primary organ involved in the metabolism of xenobiotics, assessing its function is critical in recognizing the potential toxicity of the therapies administered. Albumin is a significant biomarker of liver function, hypoalbuminemia could be caused by the liver producing less, losing more, or increasing proteolysis and clearance. ALB and A/G showed a decreasing trend in male and female rats at high doses, while ALT and AST did not show abnormalities and no albumin was detected in the urine. No abnormalities in the gastrointestinal tract, liver or kidney were found in the pathological examination. In addition, the changes were unrelated to dose and were within the laboratory's historical standard range of controls. These findings show that rats administered 8 g/kg of GLA did not experience liver or renal damage. Therefore, feeding GLA at 8 g/kg/d was not responsible for the decrease in serum levels of ALB and A/G. The liver plays a central role in detoxification. Female CK, ALT, and AST levels have decreased, which could be attributed to the presence of triterpenes and polysaccharides in GLA (Hu et al., 2020; Oluwafemi Adetuyi et al., 2020; Tong et al., 2020).

Increasing in creatinine is typically associated with visible damage to functional nephrons, which is a good indicator of renal function. There were statistically significant differences in CREA in 4 g/kg GLA‐treated animals when compared with the control group. The predominant histological findings of the kidney in the rats at 4 g/kg were normal glomerular size and number, with no tubular abnormalities. These findings suggested that the renal function might not be damaged by GLA at 4 g/kg. The changes in CL^−^ in the female groups were not considered treatment‐related because they showed no apparent dose dependence, and they were also within the normal range of laboratory reference data.

In relative organ weight, the changes in kidney relative organ weight were likewise inconsistent between the sexes and did not follow any dose‐related patterns. Additionally, no kidney histological abnormalities were found. Therefore, the slight reduction in kidney weight observed in males at 8 g/kg was not deemed harmful. In the histopathological examination, all of these pathological alterations were intermittently found during the histological analysis in both the controls and the GLA‐administered rats; however, neither sex consistently displayed any of these changes. As a result, these lesions may be regarded as spontaneous or accidental in origin but unrelated to the therapy of GLA.

## 5 CONCLUSIONS

The results of an acute study showed that the oral median lethal dose (LD_50_) of the alcoholic extract from *G. leucocontextum* is more than 16 g/kg body weight. The subacute toxicity test, which was orally administered with 2, 4, and 8 g/kg doses of GLA for 28 days, revealed no significant organ and tissue changes, the no‐observed‐adverse‐effect level (NOAEL) for GLA was 8 g/kg/day. The NOAEL level of GLA in the present study is equated to an intake for humans of 8.6 g/kg (raw material), which is 43 times the recommended daily allowance for adults. According to the study, *G. leucocontextum* was deemed safe for medical use. This research extended the *G. leucocontextum'*s safe range, served as a springboard for later studies on the activity of the plant's various medicinal parts, and established a theoretical foundation for *G. leucocontextum* in the homologies of medicine and food, as well as a scientific foundation for future research.

## FUNDING INFORMATION

This work was supported by Major entrusted research project in 2019 from Tibet Education Department, Genuine ethnic medicinal materials in Tibet‐Research on the development of the whole industrial chain of *Ganoderma leucocontextum* (Project number 2020QT022), Major science and technology research project in 2021 from Tibet Science and Technology Department, Research on the product transformation of Tibetan genuine medicinal material (Tibetan Fritillaria Bulb) in the treatment of the chronic obstructive pulmonary disease (COPD) (Project number XZ202101ZD0021G), Special fund for strategic cooperation between Sichuan University and Dazhou Municipal Government (No. 2021CDDZ‐13) and Strategic cooperation project of Sichuan University and Luzhou Municipal People's Government (No. 2019CDLZ‐25).

## CONFLICT OF INTEREST

The authors declare that this research was conducted in the absence of any commercial or financial relationships that could be construed as a potential conflict of interest.

## ETHICS STATEMENT

All the animal experiments were carried out at West China School of Pharmacy Sichuan University. All experiments were conducted in accordance with the principles of the Guide for the Care of Laboratory Animals and were approved by the Institutional Animal Ethics Committee (IAEC) of Sichuan University (license number: SYXK [Chuan] 2018–113). This study followed the National Institutes of Health's Guide for the Care and Use of Laboratory Animals to the code. All of the operations were done under sodium pentobarbital anesthesia, and every attempt was made to reduce pain.

## INFORMED CONSENT

All the listed authors have read and approved the submitted manuscript.

## Supporting information


Tables S1–S7

## Data Availability

Research data are not shared.
