# Supplementary material for: Evaluation of the acute toxicity and 28‐days subacute toxicity of the alcoholic extract from Ganoderma leucocontextum
Source: Food Sci Nutr. 2022 Sep 25;11(1):434–42. doi: 10.1002/fsn3.3075 (PMC9834818; doi:10.1002/fsn3.3075)
Supplement: Supplementary file 1 — Tables S1–S7 [file FSN3-11-434-s001.docx]

**Table S1**

LC-MS profile of the alcoholic extract from *Ganoderma leucocontextum.*

| Name | RT [min] | Formula | Molecular Weight |
| --- | --- | --- | --- |
| 3-Deoxy-D-manno-octulosonic acid | 1.01 | C8 H14 O8 | 238.06928 |
| Diacetylphloroglucinol | 1.22 | C10 H10 O5 | 210.05372 |
| 4-(5-Formyl-2-furyl)-2-hydroxybenzoic acid | 1.62 | C12 H8 O5 | 232.03715 |
| (+)-Bakuchiol | 2.89 | C18 H24 O | 256.18282 |
| Methyl 3,4,5-trimethoxycinnamate | 4.22 | C13 H16 O5 | 252.10017 |
| Asperulosidic acid | 4.52 | C18 H24 O12 | 432.1263 |
| β-D-GlcpA-(1->3)-α-D-GalpA-(1->2)-L-Rhap | 4.92 | C18 H28 O17 | 516.13013 |
| Maraniol | 5.32 | C12 H12 O3 | 204.07876 |
| (3E)-3-Benzylidene-1,1'-bi(cyclopentyl)-2-one | 5.77 | C17 H20 O | 240.15155 |
| 11alpha-hydroxy-2,3,4,5-tetranor-9,15-dioxoprostan-1,20-dioic acid | 6.43 | C16 H24 O7 | 328.15255 |
| 9,10-Dihydroxystearic acid | 7.28 | C18 H36 O4 | 316.26158 |
| Antcin B | 7.94 | C29 H40 O5 | 468.28811 |
| Podecdysone B | 8.54 | C27 H42 O6 | 462.29723 |
| Valtrate | 9.00 | C22 H30 O8 | 422.19579 |
| 16-Hydroxyhexadecanoic acid | 9.70 | C16 H32 O3 | 272.23564 |
| Estradiol cypionate | 10.30 | C26 H36 O3 | 396.26603 |
| (+/-)-Rosmarinic acid | 10.76 | C18 H16 O8 | 360.08488 |
| 5,5-Bis(methylperoxy)nonane | 12.01 | C11 H24 O4 | 220.16799 |
| 2-Hydroxyethyl 12-hydroxyoctadecanoate | 12.16 | C20 H40 O4 | 344.29274 |
| Dihydrocucurbitacin B | 13.07 | C32 H48 O8 | 560.33418 |
| Ganoderic acid Y | 13.68 | C30 H46 O3 | 454.34523 |
| Kadsurin | 14.33 | C25 H30 O8 | 458.19373 |
| 2-Octadecylfuran | 14.89 | C22 H40 O | 320.30811 |
| 10,16-Dihydroxyhexadecanoic acid | 15.54 | C16 H32 O4 | 288.23082 |
| 11-Oxoganoderiol D | 16.56 | C30 H46 O6 | 502.3272 |
| Leucocontextin U | 16.96 | C30 H50 O6 | 506.35829 |
| Methyl Linolenate | 17.37 | C19 H32 O2 | 292.23995 |
| Myristic acid | 17.77 | C14 H28 O2 | 228.37092 |
| Stearic acid | 18.07 | C18 H36 O2 | 284.47724 |
| Pentacosylic acid | 18.48 | C25 H50 O2 | 382.38223 |
| 2-hydroxycerotic acid | 18.83 | C26 H52 O3 | 412.39294 |
| 3,4-Dihydrocadalene | 20.10 | C15 H20 | 200.15659 |
| Macrocarpal B | 20.25 | C28 H40 O6 | 472.28099 |
| Nonacosylic acid | 20.81 | C29 H58 O2 | 438.44491 |
| Melissic acid | 21.27 | C30 H60 O2 | 452.46081 |
| Enrasentan | 22.13 | C29 H30 O8 | 506.19636 |
| (+)-Eudesmin | 22.63 | C22 H26 O6 | 386.17267 |
| 3-hexaprenyl-4,5-dihydroxybenzoic acid | 22.88 | C37 H54 O4 | 562.40215 |
| Hexyl cinnamaldehyde | 23.64 | C15 H20 O | 216.15146 |
| Ganoleucoin Y | 24.60 | C36 H50 O12 | 674.32989 |
| Ximenic acid | 24.75 | C26 H50 O2 | 394.38243 |

**Table S2**

Mean body weight of male and female mice treated with the alcoholic extract from *Ganoderma leucocontextum* during an acute toxicological test.

|  | Males | | Females | |
| --- | --- | --- | --- | --- |
| Body weight(g) | Control | GLA (16g/kg/day) | Control | GLA (16g/kg/day) |
| Initial weight | 27.76 ± 2.29 | 27.95 ± 2.26 | 25.42 ± 1.96 | 25.44 ± 2.02 |
| Day 2 weight | 28.98 ± 2.09 | 29.20 ± 2.21 | 25.81 ± 1.88 | 25.83 ± 1.91 |
| Day 4 weight | 30.25 ± 2.16 | 30.65 ± 2.49 | 26.33 ± 1.70 | 26.28 ± 1.80 |
| Day 6 weight | 32.02 ± 2.45 | 32.44 ± 2.74 | 26.84 ± 1.77 | 26.79 ± 1.70 |
| Day 8 weight | 34.16 ± 2.68 | 34.71 ± 3.26 | 27.63 ± 1.66 | 27.70 ± 1.68 |
| Day 10 weight | 35.40 ± 2.73 | 35.94 ± 3.37 | 28.27 ± 1.55 | 28.02 ± 1.73 |
| Day 12 weight | 36.48 ± 2.63 | 37.08 ± 3.39 | 28.73 ± 1.52 | 28.49 ± 1.57 |
| Day 14 weight | 37.67 ± 2.91 | 38.34 ± 3.41 | 29.51 ± 1.66 | 29.19 ± 1.78 |

The values are expressed as means ± standard deviation (10 rats/sex/group).

No significant differences between the control and treatment groups.

**Table S3**

Hematological parameters of mice treated orally with the alcoholic extract from *Ganoderma leucocontextum* in an acute toxicity test.

|  | | Males | | | Females | |
| --- | --- | --- | --- | --- | --- | --- |
| Hematological parameters | Control | | GLA (16g/kg/day) | Control | | GLA (16g/kg/day) |
| HGB (g/L) | 153.30 ± 8.49 | | 152.40 ± 6.54 | 151.50 ± 5.17 | | 153.10 ± 3.44 |
| WBC (×10^9^/L) | 4.77 ± 1.81 | | 4.39 ± 1.40 | 6.54 ± 2.19 | | 5.53 ± 2.13 |
| RBC (×10^12^/L) | 8.51 ± 1.16 | | 8.98 ± 0.47 | 8.20 ± 1.66 | | 9.05 ± 0.33 |
| PLT (×10^9^/L) | 991.3 ± 142.47 | | 916.20 ± 184.77 | 764.90 ± 70.43 | | 763.90 ± 79.76 |
| HCT (L/L) | 44.76 ± 5.67 | | 48.79 ± 4.63 | 45.52 ± 9.98 | | 49.18 ± 3.53 |
| MCV (fL) | 52.81 ± 4.00 | | 54.25 ± 3.78 | 55.29 ± 2.89 | | 54.33 ± 3.92 |
| MCH (pg) | 17.06 ± 0.25 | | 17.01 ± 0.39 | 17.20 ± 0.29 | | 16.93 ± 0.33 |

The values are expressed as means ± standard deviation (10 rats/sex/group).

No significant differences between the control and treatment groups.

**Table S4**

Serum biochemistry of mice treated orally with the alcoholic extract from *Ganoderma leucocontextum* in an acute toxicity test.

|  | Males | | Females | |
| --- | --- | --- | --- | --- |
| Hematological parameters | Control | GLA (16g/kg/day) | Control | GLA (16g/kg/day) |
| Males |  |  |  |  |
| ALB (g/L) | 27.67 ± 2.08 | 25.13 ± 1.46 | 31.98 ± 1.44 | 31.58 ±1.53 |
| ALT (U/L) | 59.10 ± 13.67 | 60.50 ± 8.30 | 72.90 ± 14.49 | 71.20 ± 14.86 |
| AST (U/L) | 267.10 ± 36.88 | 272.70 ± 37.96 | 270.90 ± 60.97 | 268.80 ± 65.00 |
| UREA (mmol/L) | 8.54 ± 1.26 | 8.30 ± 1.38 | 6.87 ± 1.16 | 7.78 ± 1.47 |
| CREA (µmol/L) | 7.83 ± 0.94 | 7.81 ± 0.21 | 4.70 ± 1.96 | 4.73 ± 1.75 |
| ALP (U/L) | 123.00 ± 22.40 | 145.70 ± 40.06 | 163.80 ± 43.99 | 147.30 ± 30.88 |
| CHOL (mmol/L) | 3.14 ± 0.44 | 3.22 ± 0.48 | 2.27 ± 0.43 | 2.39 ± 0.33 |

The values are expressed as means ± standard deviation (10 rats/sex/group).

No significant differences between the control and treatment groups.

**Table S5**

Hematological parameters of Sprague-Dawley rats treated orally with the alcoholic extract from *Ganoderma leucocontextum* for 28 days.

| GLA-treated groups (g/kg/body weight/d) | | | | |
| --- | --- | --- | --- | --- |
| Hematological parameters | Control | Low-dose(2g/kg/day) | Mid-dose(4g/kg/day) | High-dose(8/kg/day) |
| Males |  |  |  |  |
| HGB (g/L) | 153.50 ± 8.07 | 145.40 ± 10.56 | 158.00 ± 9.15 | 152.00 ± 7.33 |
| WBC (×10^9^/L) | 8.48 ± 1.91 | 10.19 ± 2.39 | 12.80 ± 3.05* | 8.90 ± 3.22 |
| RBC (×10^12^/L) | 7.43 ± 0.45 | 7.17 ± 0.66 | 7.85 ± 0.42 | 7.40 ± 0.38 |
| PLT (×10^9^/L) | 788.10 ± 189.75 | 734.50 ± 169.69 | 890.30 ± 341.58 | 811.00 ± 269.13 |
| LPR (%) | 81.53 ± 3.17 | 79.81 ± 5.99 | 84.40 ± 3.44 | 76.73 ± 8.24 |
| MPR (%) | 9.26 ± 1.58 | 8.48 ± 3.15 | 7.61 ± 1.70 | 9.01 ± 2.41 |
| HCT (L/L) | 44.77 ± 2.35 | 41.83 ± 3.09 | 45.27 ± 3.01 | 43.17 ± 2.40 |
| PT (s) | 16.46 ± 2.30 | 19.26 ± 2.78 | 19.69 ± 2.51 | 20.47 ± 2.43 |
| APTT (s) | 48.85 ± 13.24 | 49.08 ± 11.21 | 45.04 ± 8.68 | 42.93 ± 12.93 |
| MCV (fL) | 60.28 ± 1.42 | 59.47 ± 1.51 | 59.59 ± 1.13 | 59.85 ± 1.25 |
| MCH (pg) | 20.67 ± 0.29 | 20.51 ± 0.84 | 20.45 ± 0.56 | 20.22 ± 0.38 |
| MCHC (g/L) | 343.20 ± 4.76 | 341.22 ± 4.15 | 344.30 ± 3.16 | 344.00 ± 3.06 |
| RDWR (%) | 14.28 ± 0.73 | 14.50 ± 0.43 | 14.63 ± 0.39 | 14.48 ± 0.42 |
| RDWA (fL) | 28.33 ± 0.59 | 28.12 ± 1.19 | 29.07 ± 0.83 | 28.72 ± 0.90 |
| PCT (%) | 0.45 ± 0.11 | 0.431 ± 0.02 | 0.46 ± 0.02 | 0.45 ± 0.02 |
| MPV (fL) | 5.72 ± 0.27 | 5.69 ± 0.23 | 5.81 ± 0.27 | 5.71 ± 0.16 |
| PDW (%) | 7.93 ± 0.26 | 7.89 ± 0.17 | 8.01 ± 0.20 | 7.82 ± 0.06 |
| P-LCR (%) | 3.09 ± 0.69 | 3.50 ± 1.09 | 3.59 ± 0.81 | 2.96 ± 0.62 |
| Females |  |  |  |  |
| HGB(g/L) | 153.30 ± 6.80 | 148.60 ± 3.92 | 154.60 ± 8.07 | 153.30 ± 5.17 |
| WBC(×10^9^/L) | 9.09 ± 2.53 | 8.03 ± 1.53 | 8.66 ± 3.04 | 10.27 ± 1.45 |
| RBC(×10^12^/L) | 7.56 ± 0.35 | 7.27 ± 0.23 | 7.70 ± 0.51 | 7.71 ± 0.28 |
| PLT(×10^9^/L) | 743.30 ± 130.10 | 922.00 ± 104.44 | 875.30 ± 372.74 | 1038.20 ± 91.71* |
| LPR (%) | 85.60 ± 4.09 | 85.19 ± 3.30 | 78.76 ± 10.77* | 84.82 ± 3.87 |
| MPR (%) | 7.5 ± 1.46 | 7.31 ± 1.69 | 7.5 ± 2.99 | 6.89 ± 2.41 |
| HCT (L/L) | 44.47 ± 2.43 | 42.73 ± 1.07 | 44.30 ± 2.59 | 44.04 ± 2.02 |
| PT (s) | 20.93 ± 7.15 | 21.31 ± 8.60 | 20.30 ± 6.69 | 16.45 ± 1.28 |
| APTT (s) | 51.55 ± 10.73 | 55.50 ± 15.72 | 43.21 ± 7.22 | 43.95 ± 5.11 |
| MCV (fL) | 58.79 ± 1.81 | 58.55 ± 1.71 | 57.90 ± 1.18 | 57.56 ± 0.73 |
| MCH (pg) | 19.77 ± 0.35 | 19.95 ± 0.78 | 19.87 ± 0.49 | 19.97 ± 0.60 |
| MCHC (g/L) | 345.00 ± 4.14 | 341.90 ± 4.53 | 345.50 ± 2.59 | 345.43 ± 3.60 |
| RDWR (%) | 14.07 ± 0.70 | 13.90 ± 0.40 | 14.18 ± 0.66 | 14.34 ± 0.51 |
| RDWA (fL) | 27.24 ± 0.74 | 27.04 ± 1.09 | 26.83 ± 1.02 | 27.20 ± 0.77 |
| PCT (%) | 0.47 ± 0.05 | 0.48 ± 0.02 | 0.47 ± 0.01 | 0.48 ± 0.02 |
| MPV (fL) | 5.76 ± 0.17 | 5.65 ± 0.16 | 5.70 ± 0.24 | 5.66 ± 0.11 |
| PDW (%) | 7.94 ± 0.12 | 7.93 ± 0.12 | 7.98 ± 0.13 | 7.89 ± 0.12 |
| P-LCR (%) | 3.46 ± 0.78 | 3.10 ± 0.45 | 3.25 ± 0.16 | 3.23 ± 0.58 |

The values are presented as mean ± standard deviation (10 rats/sex/group).

*p < 0.05 compared with the control group; **p < 0.01 compared with the control group.

**Table S6**

Blood biochemical findings of female Sprague–Dawley rats treated orally the alcoholic extract from *Ganoderma leucocontextum* for 28 days.

| GLA-treated groups (g/kg/body weight/d) | | | | |
| --- | --- | --- | --- | --- |
| Hematological parameters | Control | Low-dose(2g/kg/day) | Mid-dose(4g/kg/day) | High-dose(8/kg/day) |
| Males |  |  |  |  |
| ALT (U/L) | 38.00 ± 8.07 | 39.90 ± 4.98 | 50.90 ± 16.66 | 49.60 ± 10.57 |
| AST (U/L) | 120.20 ± 11.82 | 115.90 ± 22.19 | 122.00 ± 23.76 | 120.90 ± 28.16 |
| TP (g/L) | 62.65 ± 2.24 | 61.30 ± 5.05 | 64.85 ± 5.74 | 58.54 ± 4.90 |
| ALB (g/L) | 40.04 ± 1.58 | 37.38 ± 2.40 | 39.43 ± 2.57 | 35.82 ± 2.58* |
| UREA (mmol/L) | 4.61 ± 0.89 | 4.80 ± 0.54 | 4.86 ± 1.11 | 4.84 ± 0.70 |
| CREA (µmol/L) | 20.83 ± 1.93 | 19.50 ± 1.76 | 25.33 ± 3.92* | 21.02 ± 3.73 |
| UA (µmol/L) | 69.00 ± 5.80 | 62.17 ± 12.27 | 75.84 ± 12.69 | 67.56 ± 15.71 |
| GLU (mmol/L) | 5.22 ± 0.65 | 6.14 ± 0.94 | 6.81 ± 1.04 | 6.47 ± 0.98 |
| CHOL (mmol/L) | 1.62 ± 0.26 | 1.70 ± 0.15 | 1.84 ± 0.31 | 1.74 ± 0.27 |
| TG (mmol/L) | 0.86 ± 0.44 | 0.70 ± 0.27 | 0.82 ± 0.45 | 0.85 ± 0.33 |
| ALP (U/L) | 264.50 ± 59.38 | 274.40 ± 68.29 | 312.50 ± 31.72 | 310.80 ± 47.83 |
| GGT (U/L) | 1.60 ± 0.70 | 1.50 ± 0.71 | 2.00 ± 1.76 | 1.70 ± 0.67 |
| CK (U/L) | 907.70 ± 326.85 | 1101.50 ± 535.90 | 1450.00 ± 1284.25 | 1272.90 ± 1023.43 |
| TBIL (µmol/L) | 0.89 ± 0.19 | 0.70 ± 0.13 | 0.74 ± 0.23 | 0.80 ± 0.08 |
| DBIL (µmol/L) | 0.61 ± 0.12 | 0.47 ± 0.16 | 0.59 ± 0.18 | 0.65 ± 0.10 |
| A/G | 1.78 ± 0.12 | 1.61 ± 0.29 | 1.60 ± 0.29 | 1.61 ± 0.23 |
| K^+^(mmol/L) | 6.36 ± 0.46 | 6.56 ± 0.58 | 6.842 ± 0.60 | 6.88 ± 0.64 |
| Na^+^(mmol/L) | 143.91 ± 2.11 | 142.83 ± 2.46 | 146.71 ± 3.77 | 143.41 ± 2.30 |
| CL^-^(mmol/L) | 99.80 ± 1.38 | 101.01 ± 2.00 | 102.73 ± 2.05 | 101.31 ± 1.94 |
| Females |  |  |  |  |
| ALT (U/L) | 44.00 ± 6.21 | 30.50 ± 5.70* | 37.20 ± 10.44 | 42.50 ± 9.28 |
| AST (U/L) | 129.00 ± 21.93 | 110.00 ± 14.85 | 118.90 ± 24.41 | 108.10 ± 12.99 |
| TP (g/L) | 65.94 ± 2.55 | 63.94 ± 3.14 | 68.74 ± 6.03 | 64.82 ± 1.56 |
| ALB (g/L) | 42.09 ± 2.41 | 41.39 ± 1.69 | 42.02 ± 3.57 | 39.24 ± 0.89* |
| UREA (mmol/L) | 6.94 ± 0.65 | 6.38 ± 0.66 | 9.09 ± 4.36 | 6.11 ± 1.06 |
| CREA (µmol/L) | 23.90 ± 2.11 | 26.52 ± 2.03 | 29.11 ± 6.62 | 26.31 ± 2.85 |
| UA (µmol/L) | 73.85 ± 11.48 | 62.53 ± 11.87 | 72.46 ± 23.61 | 74.20 ± 16.68 |
| GLU (mmol/L) | 6.35 ± 0.53 | 6.52 ± 0.72 | 6.45 ± 0.42 | 6.43 ± 0.63 |
| CHOL (mmol/L) | 2.05 ± 0.30 | 2.07 ± 0.38 | 1.95 ± 0.37 | 2.38 ± 0.32 |
| TG (mmol/L) | 0.59 ± 0.31 | 0.48 ± 0.13 | 0.45 ± 0.38 | 0.43 ± 0.17 |
| ALP (U/L) | 145.10 ± 22.76 | 155.50 ± 40.35 | 176.30 ± 33.09 | 169.00 ± 22.94 |
| GGT (U/L) | 1.40 ± 0.60 | 1.20 ± 0.42 | 1.70 ± 0.94 | 1.40 ± 0.51 |
| CK (U/L) | 2127.20 ± 907.93 | 1511.50 ± 665.66 | 1316.90 ± 786.94 | 1110.40 ± 365.81* |
| TBIL (µmol/L) | 0.72 ± 0.17 | 0.68 ± 0.12 | 0.52 ± 0.19 | 0.70 ± 0.15 |
| DBIL (µmol/L) | 0.59 ± 0.17 | 0.43 ± 0.11 | 0.53 ± 0.21 | 0.55 ± 0.13 |
| A/G | 1.79 ± 0.20 | 1.85 ± 0.11 | 1.60 ± 0.22 | 1.53 ± 0.08* |
| K^+^ (mmol/L) | 7.14 ± 0.57 | 6.53 ± 0.59 | 7.32 ± 0.55 | 6.74 ± 0.42 |
| Na^+^ (mmol/L) | 143.28 ± 2.23 | 143.13 ± 1.56 | 141.47 ± 4.10 | 144.40 ± 1.99 |
| CL^-^ (mmol/L) | 100.23 ± 1.66 | 100.59 ± 1.65 | 99.89 ± 2.89 | 104.56 ± 2.44* |

The values are expressed as mean ± standard deviation (10 rats/sex/group).

*p < 0.05 compared with the control group; **p < 0.01 compared with the control group.

**Table S7**

Effect of subacute administration of the alcoholic extract from *Ganoderma leucocontextum* on terminal body weight and organic coefficient (g/kg) in grams of male and female rats.

| GLA-treated groups (g/kg/body weight/d) | | | | |
| --- | --- | --- | --- | --- |
| Relative organ weight | Control | Low-dose(2g/kg/day) | Mid-dose(4g/ka/day) | High-dose(8/kg/day) |
| Males |  |  |  |  |
| Liver | 31.49 ± 2.92 | 34.22 ± 4.28 | 34.83 ± 2.60 | 34.02 ± 3.97 |
| Brain | 5.85 ± 0.94 | 5.10 ± 0.78 | 5.89 ± 0.93 | 4.92 ± 0.27 |
| Heart | 3.68 ± 0.38 | 4.07 ± 0.41 | 3.44 ± 0.47 | 3.59 ± 0.33 |
| Thymus | 2.17 ± 0.27 | 2.49 ± 0.45 | 2.45 ± 0.36 | 2.46 ± 0.39 |
| Lungs | 5.27 ± 0.93 | 6.81 ± 0.81 | 5.92 ± 1.59 | 6.47 ± 1.17 |
| Spleen | 2.63 ± 0.30 | 3.15 ± 0.46 | 2.66 ± 0.39 | 2.63 ± 0.45 |
| Kidneys | 6.67 ± 1.36 | 7.93 ± 0.82 | 7.58 ± 0.61 | 8.07 ± 0.56* |
| Adrenal glands | 0.19 ± 0.05 | 0.24 ± 0.06 | 0.19 ± 0.03 | 0.20 ± 0.04 |
| Testes | 9.04 ± 1.62 | 9.78 ± 1.99 | 10.27 ± 0.99 | 9.95 ± 0.50 |
| Females |  |  |  |  |
| Liver | 35.91 ± 5.10 | 33.7 ± 4.52 | 38.22 ± 7.87 | 38.73 ± 3.49 |
| Brain | 7.08 ± 1.20 | 5.90 ± 0.44 | 6.60 ± 0.78 | 6.56 ± 0.83 |
| Heart | 3.92 ± 0.54 | 3.87 ± 0.74 | 3.61 ± 0.24 | 3.8 ± 0.36 |
| Thymus | 2.51 ± 0.31 | 2.70 ± 0.20 | 2.72 ± 0.19 | 2.79 ± 0.21 |
| Lungs | 7.07 ± 0.74 | 7.12 ± 0.88 | 7.73 ± 1.10 | 7.18 ± 1.37 |
| Spleen | 2.68 ± 0.60 | 2.65 ± 0.58 | 2.54 ± 0.36 | 2.69 ± 0.21 |
| Kidneys | 8.39 ± 1.24 | 8.01 ± 1.20 | 8.19 ± 0.51 | 8.05 ± 0.63 |
| Adrenal glands | 0.42 ± 0.37 | 0.40 ± 0.10 | 0.34 ± 0.04 | 0.33 ± 0.07 |
| Ovary | 2.88 ± 0.58 | 2.77 ± 0.53 | 2.80 ± 0.48 | 2.71 ± 0.37 |

The values are expressed as mean ± standard deviation (10 rats/sex/group).

*p < 0.05 compared with the control group; **p < 0.01 compared with the control group.
